# A Rare Case of Malignant Tumor of the Larynx with Good Prognosis: Laryngeal Chondrosarcoma

**DOI:** 10.1155/2019/9468194

**Published:** 2019-05-05

**Authors:** Abderrahim Elktaibi, Issam Rharrassi, Nabil Hammoune, Youssef Darouassi, Mohammed Amine Hanine, Haddou Ammar

**Affiliations:** ^1^Department of Pathology, Avicenne Military Hospital, Marrakech, Morocco; ^2^Department of Radiology, Avicenne Military Hospital, Marrakech, Morocco; ^3^Department of Otolaryngology, Avicenne Military Hospital, Marrakech, Morocco

## Abstract

Laryngeal chondrosarcoma is a rare laryngeal tumor that most frequently originates from the cricoid cartilage. The majority of lesions are low grade and the distinction from benign chondromas must be made. We present a case of a laryngeal chondrosarcoma arising from the cricoid cartilage in a 75-year-old Arab man who presented with hoarseness, dysphonia, and dyspnea. Endoscopic and radiological examinations showed a mass of the wall of his larynx with displaced structures, airway obstruction, and destruction of the cartilage. The patient underwent total laryngectomy. Histological examination supported the diagnosis of low-grade chondrosarcoma. Five months later, the radiological and clinical findings showed no evidence of recurrence or metastases. Laryngeal chondrosarcomas remain a rare disease of unknown etiology, with slow and insidious symptoms. The treatment is surgical, given the importance of preserving the larynx to patients' quality of life. The prognosis is favorable and metastases rarely occur.

## 1. Introduction

Chondrosarcoma of the larynx is a rare tumor representing about 1% of all laryngeal neoplasms [1]. It was first described by Travers in 1816 [2], but the term chondrosarcoma at this site was introduced by New in 1935 [3]. The diagnosis requires clinical, histological, and radiographic correlation. It typically exhibits low-grade histology, with fairly indolent progression and a relatively low probability of metastatic potential or recurrence. For this reason, conservative surgical excision with negative margins is recommended [4]. We present a case of laryngeal chondrosarcoma from the cricoid cartilage in a patient who presented with hoarseness and dyspnea and cervicofacial computed tomography scan, revealing a large tumor requiring total laryngectomy.

## 2. Case Presentation

**Figure 1:**
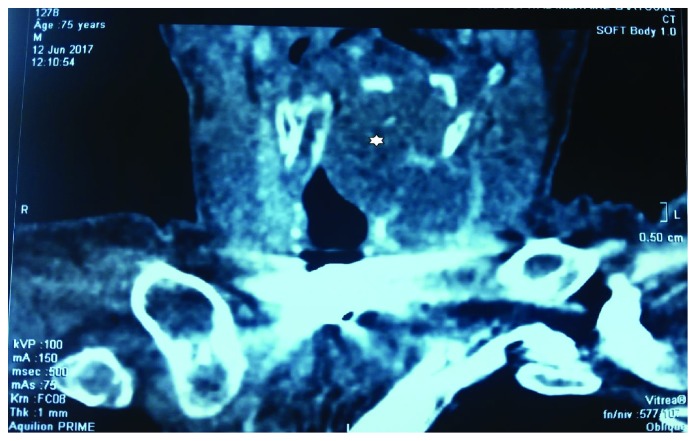
Computed tomography of the head and neck showed a subglottic mass containing calcifications and causing airway obstruction (star).

**Figure 2:**
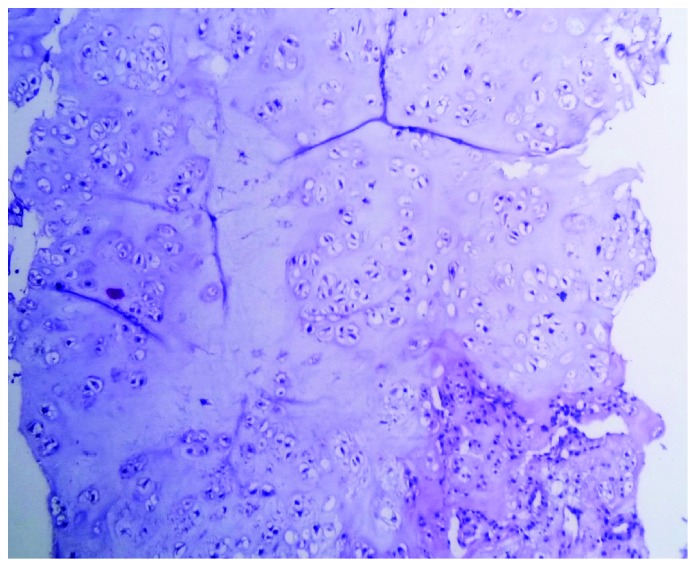
Mildly cellular field with predominantly small mono- and binucleated chondrocytes with ample cytoplasm, consistent with low grade (grade 1). Haematoxylin & eosin staining; medium power magnification ×250.

**Figure 3:**
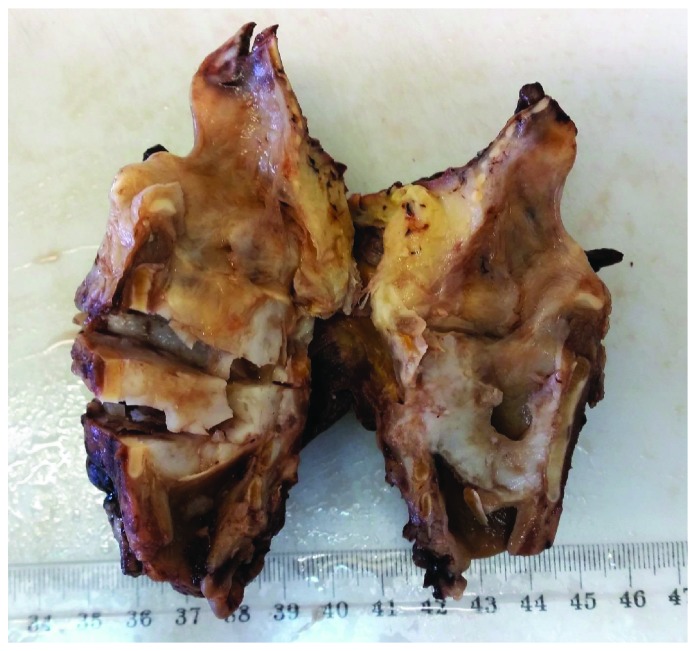
Macroscopic appearance of the laryngectomy showing a translucent tumor of the larynx.

A 75-year-old Arab male, chronic smoker, presented with a history of progressive dysphonia, hoarseness, airway obstruction, and worsening odynophagia over a 6-month period. The direct laryngoscopic test showed a left paramedian glottic and subglottic tumefaction, surrounded by intact mucosa and fixed homolateral hemilarynx. No adenopathy was found in the laterocervical region. The patient underwent neck computed tomography (CT) which showed a large mass of glottic and subglottic plan lateralized on the left measuring 5 × 3.5 cm containing calcifications, causing a retraction of the laryngeal diameter and destruction of the cricoid cartilage (Figure 1). No infiltration of adjacent surgical plans and no adenopathies were detected. Gross pathology from a biopsy of mass consisted of numerous fragments of soft tissue, with firm consistency, roughened, and semitranslucent cut surfaces. Histological examination of the biopsy showed hyaline cartilage comprised of lobules of binucleated chondrocytes with increased nucleus to cytoplasmic ratios (Figure 2). Mitotic activity was not identified. There was no evidence of a myxoid matrix and any areas of necrosis. These findings are diagnostic of a low-grade (grade 1 of 3) chondrosarcoma of the larynx. On the basis of the histological and radiological examinations performed, the patient underwent total laryngectomy (Figure 3). The final histological examination confirmed the diagnosis of a well-differentiated grade 1 chondrosarcoma of the larynx. The patient was followed up for 5 months without any sign of recurrence or metastases.

## 3. Discussion

Laryngeal chondrosarcoma is a rare tumor, accounting for less than 1% of all sarcomas of the body. The tumor most commonly occurs from the cricoid cartilage and less frequently from the arytenoid and thyroid cartilage [5]. The etiology of laryngeal chondrosarcoma is imprecise, but it seems to result from disordered ossification of the laryngeal cartilage [6]. The current data do not show a causal relationship with smoking or alcohol consumption [1]. The mean age at diagnosis is 60 to 64 years, with a male predominance [7]. The symptoms are variable depending on the location of the mass and include hoarseness (most patients) caused by narrowing of the glottic plane and compression of the inferior laryngeal nerves; dyspnea and airway obstruction due to endolaryngeal growth; dysphagia due to extralaryngeal growth, originating in the posterior cricoid; and neck mass due to tumor involvement of the thyroid cartilage [6]. Imaging, including CT, MRI, or X-ray, has a major contribution for the suggestion of a chondroid neoplasm. An expansive lesion with variable density and characteristic “popcorn” calcifications is usually observed [1]. The majority of laryngeal chondrosarcomas are histologically low grade, and imaging does not always definitively differentiate a grade 1 chondrosarcoma from a benign chondroma, knowing that these two lesions can be associated with 62% of cases [4]. Invasion into adjacent soft tissue or bone may be present. Macroscopically, tumor ranges in size from less than 1 cm to up to 10 cm. The specimen is often received fragmented and it consistently shows smooth, lobular, and translucent tissue portions [5]. The histological diagnosis is based on the criteria for cartilaginous malignancies reported by Lichtenstein and Jaffe in 1943 [8]. There are three different grades of chondrosarcoma:

- Grade 1: similar to a chondroma, more than two nuclei, no mitoses, some areas of calcification and actual bone tissue (70–80% of cases)
- Grade 2: increase in cell number, low nuclear/cytoplasmic ratio, scarce mitoses (10–15% of cases)
- Grade 3: multinucleated cells, increased nuclear/cytoplasmic ratio, high number of mitoses (5–10% of cases)

About 80% of laryngeal chondrosarcomas are low grade and are usually well differentiated with a less aggressive pattern compared to chondrosarcoma at the more common sites, mainly the pelvis, long bones, sternum, and ribs [9]. The initial treatment of chondrosarcoma of the larynx is surgery [10]. The goal is to obtain total excision with negative margins [11]. For low-grade tumors, the therapeutic modalities include CO_2_ laser resection, hemicricoidectomy, or hemilaryngectomy. For high-grade tumors and recurrences, the treatment of choice is total laryngectomy. When cricoidectomy is performed, laryngotracheal anastomosis or reconstruction by flap is used to close the defect. The effectiveness of radiotherapy is still controversial. Chemotherapy has no therapeutic interest [12]. According to the data of the literature, the rate of tumor recurrence varies from 18 to 40% [1]. Nevertheless, the long-term prognosis of laryngeal chondrosarcoma is good (95%, 10-year survival) [1]. Metastatic potential is rare in grade 1 chondrosarcomas and seen in only 10% of grade 2 lesions [4]. A study of 111 cases conducted by the Armed Forces Institute of Pathology revealed that only 1.9% of cases had developed metastases [5]. For this reason, conservative therapy is the treatment of choice. Total laryngectomy is reserved for large tumors in which surgery would cause destabilization of the cricoid cartilage [6].
